# Effects of actuation of nanoporous gold on cell orientation in a fibroblast sheet

**DOI:** 10.1007/s10856-021-06584-w

**Published:** 2021-08-18

**Authors:** Peizheng Wu, Shogo Sawaki, Masataka Hakamada, Mamoru Mabuchi

**Affiliations:** Graduate School of Energy Science, Kyoto University, Yoshidahonmachi, Sakyo-ku, Kyoto, 606–8501 Japan

## Abstract

Mechanical stimulation such as flood flow often plays a vital role in the growth and maintenance of a living body, and it is important to investigate cell responses to mechanical stimulation. To date, cell responses to mechanical stimulation have been investigated in detail. However, the cell responses have been little known in a cell sheet. In the present study, a small cyclic strain (CS) of ~0.5% generated by a nanoporous gold actuator was loaded on a cell sheet of fibroblasts, and the effects of the CS on cell orientation were investigated. Individual cells were randomly distributed after the CS application, whereas cells were oriented in a specific direction after the CS application for the cell sheet. Thus, the CS had a different effect on the cell sheet from that on the individual cells. It is suggested that the cadherin/p-120 catenin complex played an important role in the cell response to mechanical stimulation in a cell sheet.

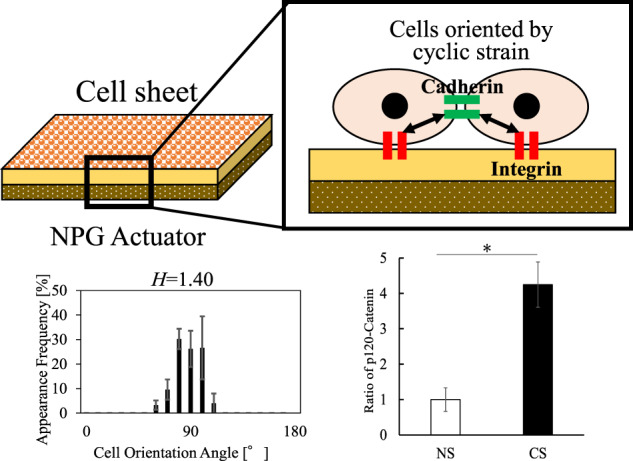

## Introduction

Mechanical stimulation affects the orientation of cells, for example, cells are oriented parallel to the loading direction by static stress [1], while they are oriented vertically to the loading direction by cyclic stress [2, 3]. It is known that integrin is important for mechanosensing [4]. It is necessary to prevent the transfer of a mechanical stimulus via molecules other than integrins to investigate the roles of integrins in mechanosensing. Scaffold materials, such as hydrogels and silicone, have been used to load a mechanical stimulus on cells [5–8]. However, the transfer of a mechanical stimulus via other molecules cannot be prevented when a scaffold is used.

A nanoporous gold (NPG) actuator has been developed using charge-induced reversible straining [9]. Because amino acids bind to gold [10], cells tend to adhere to a gold surface. Hence, a scaffold can be omitted by using a gold substrate. In the present study, cyclic strain (CS) generated by an NPG actuator substrate is loaded on a cell sheet of fibroblasts, and the effects of the CS on cell orientation are investigated. There are limited works on the effect of mechanical stimulation on a cell sheet [11]. The present work shows that the CS generated by an NPG actuator had a different effect on the cell sheet from that on the individual cells.

## Materials and methods

### Materials

The NPG actuator used in a previous work [12] was used in the present work (Fig. S1). By loading square wave (−1 ~ +1 V, 1 Hz), the NPG actuator generated 0.5% CS. Human embryo-derived fibroblasts (HEFs), which were purchased from JCRB Cell Bank, were used because the HEFs were affected by the mechanical stimulus generated by an NPG actuator [12].

### Cell experiments

All samples were cultured in DMEM with 10% fetal bovine serum and 1% Antibiotic-Antimycotic (Nacalai Tesque) at 310 K, 5% CO_2_ condition. For individual cells samples, HEFs (3.6 × 10³ cells/cm²) were seeded onto the substrates and then pre-cultured for 12 h. For cell sheets samples, HEFs (1.5 × 10^5^ cells/cm²) were seeded onto the substrates and then pre-cultured for 24 h to form a cell sheet (Fig. S2). The CS was loaded 24 h for individual cells samples and 48 h for cell sheets samples. Non-strained (NS) samples were cultured under the same conditions but without the CS.

### Cell orientation assay

After NS and CS loading, orientations of individual cells and cell sheets were characterized by Live/Dead Assay (Invitrogen) and immunofluorescence. Acti-stain^TM^ 555 Fluorescent Phalloidin (Cytoskeleton) and DAPI (Sigma) were used to visualize actin filaments and nuclei. All stained samples were observed with a BX53 fluorescence microscope (Olympus). ImageJ was used to quantify the orientations. The major axis of HEFs was measured by actin filaments, where the long axis direction of the substrate was set to 0°. The entropy of orientation angle distributions was calculated to quantitatively estimate the orientation of actin filaments. The experiments for cell orientation were carried out three times, respectively.

### Cell–cell adhesion molecule analysis

For cell sheet samples, immunofluorescence and immunoblotting of p120-catenin were performed to investigate the activated cell–cell adhesion after NS and CS loading for 48 h according to the manufacturer protocols. For immunofluorescence, anti-p120-catenin (Sigma) primary antibody and Goat Anti-Mouse Alexa Fluor 488 (Abcam) secondary antibody were used. For immunoblotting, anti-p120-catenin (Sigma) primary antibody and Goat Anti-Mouse HRP (Abcam) secondary antibody were used. The immunoblotting experiments for anti-p-120-catenin were carried out four times.

## Results

**Fig. 1:**
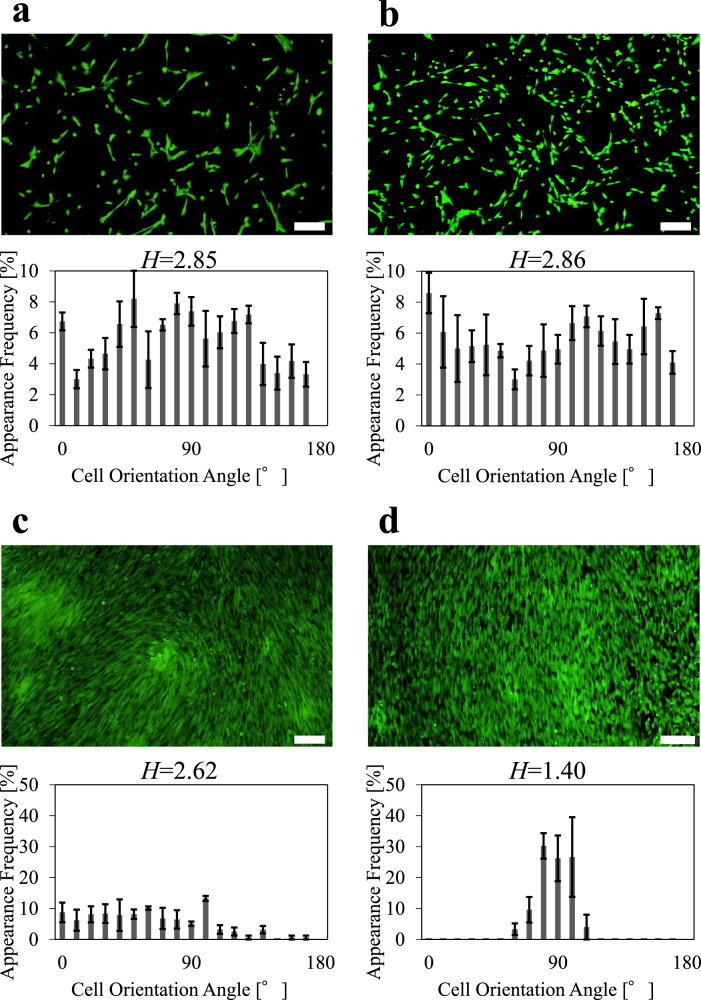
Orientation of cells. Image and quantitative analysis of individual cells for NS (**a**), individual cells for CS (**b**), cells in a cell sheet for NS (**c**), and cells in a cell sheet for CS (**d**). NS no strain, CS cyclic strain. *N* = 3. Results are shown as mean ± SE. *H* is the Shannon entropy. The scale bar is 200 μm

The orientation of cells for the individual cells and the cell sheet is shown in Fig. 1. Almost no dead cells were found after the CS application for both the individual cells and the cell sheet. Thus, the application of the voltage had no harmful effects on the cell viability. For the individual cells, the cells were not oriented in a specific direction and they were randomly distributed for NS and CS. This coincides with the results of the previous study on the NPG actuator [12]. However, the cells were oriented in a specific direction after the CS application for the cell sheet, although they were randomly distributed for NS. Thus, the cell response to CS for the cell sheet was different from that for the individual cells.

The cell orientation may be related to the cytoskeleton such as actin filaments. Hence, the orientation of actin filaments was investigated (Fig. S3). The actin filaments were not oriented in a specific direction after the CS application for the individual cells, whereas they were oriented in a specific direction after the CS application for the cell sheet. This corresponds to the result on the cell orientation. Clearly, the CS generated by the NPG actuator had a different effect on the cell sheet from that on the individual cells.

Matsugaki et al. [11] showed that mechanical stimulation affects the orientation of the extracellular matrix as well as the orientation of cells. In the present work, the secreted fibronectins were oriented in a specific direction after the CS application for the cell sheet (Fig. S4), which suggests that the cell orientation was related to the orientation of the extracellular matrix. This fact in the cell sheet agrees with the result of the work by Matsugaki et al. [11]. However, the secreted fibronectins were not oriented in a specific direction after the CS application for the individual cells. Thus, the CS had a different effect on the cell sheet from that on the individual cells in respect of the orientation of secreted fibronectins as well as the cell orientation.

**Fig. 2:**
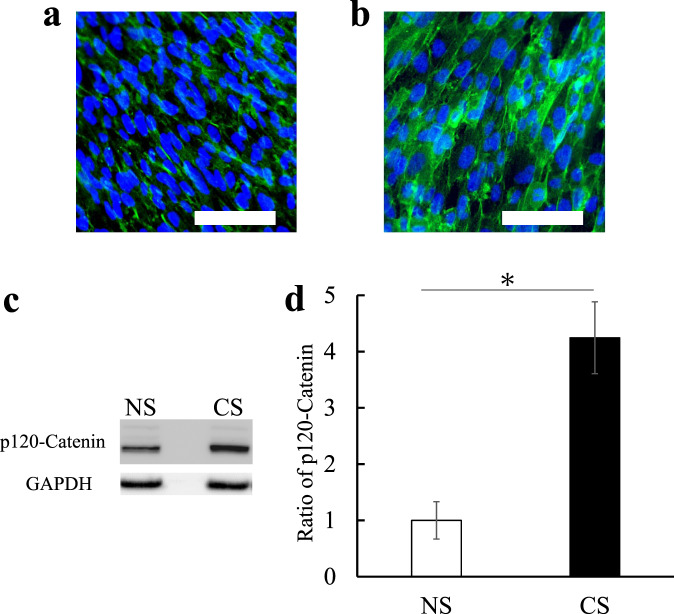
Immunofluorescence images (**a**) and (**b**) and Western blot results (**c**) and (**d**) of p120-catenin for cell sheets cultured under NS and CS conditions, (**a**) for NS and (**b**) for CS, where p120-Catenin is visualized in green, nuclei in blue. Scale bar:100 µm. In (**d**), the ratio of p120-Catenin is quantified by the Western blot with an anti-p120-Catenin antibody. *N* = 4. Results are shown as means ± SE, *p < 0.05 (**c**). NS no strain, CS cyclic strain

Cell responses often depend on the stress direction. For example, cells tend to be oriented perpendicular to the stress direction when a CS is loaded on cells [2, 3]. Also, the orientation of secreted ECM depends on the stress direction [13]. In the present study, however, the cells and secreted fibronectins were randomly distributed for the individual cells regardless of the CS application. This is because the small CS generated by the NPG actuator had a limited effect only on integrins and the cytoskeletal pre-stress state was hardly changed [12]. However, the present work showed that cells were oriented by the CS application in the cell sheet. This is suggested to be related to cell–cell adhesion. Thus, the p120 catenin, which is the cell–cell junction protein [14], was investigated for the cell sheets cultured under NS and CS conditions (Fig. 2). The results showed that the cell–cell adhesion in the cell sheet was activated by the CS application. The cadherin/p-120 catenin complex plays a constitutive role in transducing mechanical stimulation between the actomyosin cytoskeleton and the plasma membrane [15]. Therefore, it is suggested that the cell orientation by the CS application in the cell sheet is related to the transduction of mechanical stimulation through the cadherin/p-120 catenin complex.

The experiments in the present work were performed under the 0.5% CS application on the fibroblasts. More understandings about the effects of the actuation on cell orientation will be obtained by performing studies on more types of cells in the conditions of various strains, and the studies are under planning.

## Conclusions

A small CS of ~0.5% generated by an NPG actuator was loaded on a cell sheet of fibroblasts, and the effects of the CS on cell orientation were investigated. The individual cells were not oriented in a specific direction and they were randomly distributed after the CS application. However, the cells were oriented in a specific direction after the CS application for the cell sheet. This trend was found in the orientation of actin filaments and secreted fibronectins. Thus, the CS had a different effect on the cell sheet from that on the individual cells. It was suggested that the cadherin/p-120 catenin complex played an important role in cell response to mechanical stimulation in a cell sheet.

## Supplementary information

Supplementary Information

## Data Availability

The data that support the findings of this study are available from the corresponding author upon reasonable request.
