# Supplementary material for: Effects of actuation of nanoporous gold on cell orientation in a fibroblast sheet
Source: J Mater Sci Mater Med. 2021 Aug 18;32(9):103. doi: 10.1007/s10856-021-06584-w (PMC8373723; doi:10.1007/s10856-021-06584-w)
Supplement: Supplementary file 1 — Supplementary Information [file 10856_2021_6584_MOESM1_ESM.docx]

**Effects of actuation of nanoporous gold on cell orientation in a fibroblast sheet**

Peizheng Wu^*^, Shogo Sawaki, Masataka Hakamada and Mamoru Mabuchi

Graduate School of Energy Science, Kyoto University,

Yoshidahonmachi, Sakyo-ku, Kyoto 606–8501 Japan

^*^Corresponding author. Tel.: +81 75 753 5421; fax: +81 75 753 5428

E-mail address: wu.peizheng.68z@st.kyoto-u.ac.jp.

**Fig. S1** Nanoporous Au (NPG) actuator. (a) SEM image of NPG. The ligament diameter of NPG was about 15 nm. (b) performance of NPG actuator. The NPG actuator generated 0.5% strain in the tensile direction and 0.2% strain in the compression direction.

**Fig. S2** Confluent cells of a fibroblast sheet. The scale bar is 100 μm.

**Fig. S3** Orientation of actin filaments. Image and quantitative analysis of individual cells for NS (a), individual cells for CS (b), cells in a cell sheet for NS (c), and cells in a cell sheet for CS (d). NS: no strain, CS: cyclic strain. N=3. Results are shown as mean ± SE. *H* is the Shannon entropy. The scale bar is 200 μm

**Fig. S4** Orientation of fibronectins. Image and quantitative analysis of individual cells for NS (a), individual cells for CS (b), cells in a cell sheet for NS (c), and cells in a cell sheet for CS (d). NS: no strain, CS: cyclic strain. N=3. Results are shown as mean ± SE. *H* is the Shannon entropy. The scale bar is 200 μm.
